# Special Issue: “Drug Repurposing for Cancer Therapies”

**DOI:** 10.3390/ijms25021092

**Published:** 2024-01-16

**Authors:** Cristina P. R. Xavier, Andreia Palmeira

**Affiliations:** 1 i3S—Instituto de Investigação e Inovação em Saúde, Universidade do Porto, Rua Alfredo Allen 208, 4200-135 Porto, Portugal; 2 Cancer Drug Resistance Group, IPATIMUP-Institute of Molecular Pathology and Immunology of the University of Porto, Rua Alfredo Allen 208, 4200-135 Porto, Portugal; 3 LQOF—Laboratory of Organic and Pharmaceutical Chemistry, Department of Chemical Sciences, Faculty of Pharmacy, University of Porto, Rua de Jorge Viterbo Ferreira 228, 4050-313 Porto, Portugal; 4 CIIMAR—Interdisciplinary Centre of Marine and Environmental Research, Terminal de Cruzeiros do Porto de Leixões, 4450-208 Matosinhos, Portugal

Cancer is one of the primary global causes of death, thus addressing cancer therapy remains a significant challenge, especially in cases where cancers exhibit resistance to treatment [1]. To improve cancer treatment, there is a demand for novel and more effective therapeutic strategies. Drug repurposing has emerged as a strategy for the development of novel treatments using approved or investigational drugs outside the scope of their original clinical indication, being an interesting alternative to the conventional drug discovery process [2,3]. In recent years, several studies have focused on the investigation of potential drug repurposing candidates for cancer treatment, either as single agents or in combination with approved cancer therapies, by targeting multiple hallmarks of cancer [3,4,5].

The objective of this Special Issue was to gather original research articles studying the impact of drugs previously approved for non-cancer diseases on cancer treatment. The research papers published in this Issue pinpointed specific repurposed drugs that demonstrated efficacy, either alone or in combination therapy, against various types of cancer. Most of the presented papers explored the antitumor potential of clinically approved drugs by studying their effects not only in conventional two-dimensional (2D) cellular models but also in three-dimensional (3D) cell cultures designed to closely mimic the in vivo microenvironment [6].

For instance, Vineela Parvathaneni et al. described the effect of the drug amodiaquine, an old FDA (Food and Drug Administration)-approved anti-malarial drug, in breast cancer treatment [7]. The authors showed the ability of amodiaquine to induce cytotoxicity against different breast cancer cell lines. Amodiaquine was capable of inhibiting cell migration, colony formation, and vascular network formation in a dose-dependent manner. Notably, the anticancer effectiveness of this drug was confirmed in 3D spheroids. In this model, amodiaquine reduced spheroid volume, induced apoptosis, and blocked the progression of the cell cycle profile. Moreover, the authors identified the upregulation of apoptosis- and autophagy-related genes and the downregulation of genes essential for DNA transcription as mechanisms of action of amodiaquine. This work was the first report of the efficacy and the detailed molecular mechanism of action of amodiaquine for the treatment of breast cancer, although further studies are required to proceed to clinical trial.

In another approach, Yung-Lun Ni et al. reported that disulfiram, an approved drug for the treatment of alcoholism, targeted cancer stem cells in thyroid carcinoma when combined with copper [8]. Indeed, the combination of disulfiram and copper not only hindered cell proliferation but also suppressed spheroid formation and inhibited the activity of cancer stem cells in multiple differentiated thyroid carcinoma cell lines. Importantly, the authors identified the molecular mechanism involved in disulfiram/copper combination. This mechanism is associated with the downregulation of BMI1 (B lymphoma Mo-MLV insertion region 1 homolog) through the suppression of c-Myc and E2F1, as well as the decrease of cell cycle-related proteins. c-Myc is an important oncogene that contributes to carcinogenesis, being amplified in 28% of human cancers. On the other hand, E2F1 is overexpressed in many cancers, being involved in cell cycle regulation and in maintaining cancer stem cells. Hence, the authors proposed that disulfiram could emerge as a promising repurposed drug for the treatment of thyroid carcinoma when used in combination with existing treatments, especially in cases where there is overexpression of c-Myc or E2F1.

In another study, Branco et al. showed that pirfenidone, an antifibrotic drug FDA -approved for the treatment of idiopathic pulmonary fibrosis, sensitizes several non-small-cell lung cancer (NSCLC) cell lines to paclitaxel and to paclitaxel plus carboplatin treatments [9]. The combination of pirfenidone with paclitaxel was able to reduce the growth, viability, and proliferation of different NSCLC cell lines, as well as to induce major alterations in the cell cycle profile and increase cell death. Importantly, pirfenidone did not cause major cytotoxicity against human non-tumorigenic cells when compared with paclitaxel treatment alone. In the end, the authors also confirmed the capability of pirfenidone to sensitize NSCLC cells to paclitaxel plus carboplatin treatment. This was the first report to investigate the possibility of repurposing pirfenidone in combination with platinum-based regimens in vitro. Nevertheless, the authors are conscious that these findings should be validated in more complex biological models to acquire more robust preclinical data to support a clinical trial.

In the same vein, Li-Chun Liu et al. demonstrated the antitumor effect of tramadol on human endometrial cancer cells and its ability to potentiate chemotherapeutic drugs [10]. The authors showed that tramadol inhibited cell proliferation, induced cell cycle arrest, and increased reactive oxygen species generation and apoptosis in several endometrial cancer cell lines. The authors also recognized mitochondrial damage as part of the mechanism of tramadol-induced cytotoxicity. Significantly, the authors verified that tramadol had a synergistic effect when combined with doxorubicin or cisplatin in endometrial cancer cells. Thus, this work highlighted the antitumor potential of tramadol and its promising potential to be used in adjuvant therapy for endometrial cancer.

Another paper published by Keying Liang and colleagues proposed a novel combined targeted therapy for cancer patients with higher expression levels of polo-like kinase 1 (PLK1) and ATP citrate synthase (ACLY) [11]. By using RNA-seq technology, the authors verified that the combined inhibition of ubiquitin conjugating enzyme E2 C (UBE2C) and PLK1 reduced cancer cell proliferation, migration, and colony formation, while inducing cell cycle arrest and apoptosis. This strategy allowed the authors to envision novel combination therapies based on clinically approved drugs. Moreover, the authors confirmed a synergistic inhibition of cell viability in pan-cancer cell models when combining volasertib, a PLK1 inhibitor and an FDA-approved drug for the treatment of acute myeloid leukemia, with bempedoic acid, an ALCY inhibitor and an FDA-approved drug to reduce low-density lipoprotein (LDL) cholesterol levels. In conclusion, the authors suggested that targeting ACLY and PLK1 with existing drugs could offer a new option for cancer treatment, although additional preclinical studies are necessary to clarify potential clinical benefits.

This Special Issue also aimed to collect review articles about the advantages of studying repurposed drugs for cancer therapy and to raise awareness about the already repurposed drugs currently being used in the clinic to treat cancer.. The review article published in this Special Issue emphasizes the ongoing advances in personalized breast cancer treatment, stressing the importance of patient tumor heterogeneity when exploring the use of repurposed drugs and their associated advantages in this context [12]. The authors’ idea was to first cluster breast cancer patients according to their clinical and biological similarities to discover new potential targets that could allow more effective and personalized cancer therapy. This will help to select the more appropriate repurposed drugs for a more specific therapeutic regimen. In this review, the authors also described some of the repurposed drugs already used in breast cancer treatment, with an emphasis on the biomarkers related to the drug´s outcomes. From the authors’ point of view, future directions in cancer treatment concerning the applicability of drug repurposing should consider the tumor genetic profiles of each patient. This approach is seen as a potential strategy for achieving faster and more precise cancer therapies.

In summary, this Special Issue brings together several original research papers and a comprehensive review, enhancing our understanding on the advantages of investigating repurposed drugs and suggesting the potential of both single and combinatorial therapies for the treatment of various types of cancers.
